# Supplementary material for: Stacking Model‐Based Classifiers for Dealing With Multiple Sets of Noisy Labels
Source: Biom J. 2025 Mar 12;67(2):e70042. doi: 10.1002/bimj.70042 (PMC11898607; doi:10.1002/bimj.70042)
Supplement: Supplementary file 1 — Supporting Information [file BIMJ-67-e70042-s001.zip › density-based-ensemble-model-main/competitors_real_data/netlab-master/netlab-master/help/kmeans.htm]

Netlab Reference Manual kmeans


# kmeans

## Purpose

Trains a k means cluster model.

## Synopsis

```
centres = kmeans(centres, data, options)
[centres, options] = kmeans(centres, data, options)
[centres, options, post, errlog] = kmeans(centres, data, options)
```

## Description

`centres = kmeans(centres, data, options)`
uses the batch K-means algorithm to set the centres of a cluster model.
The matrix `data` represents the data
which is being clustered, with each row corresponding to a vector.
The sum of squares error function is used. The point at which
a local minimum is achieved is returned as `centres`. The
error value at that point is returned in `options(8)`.

`[centres, options, post, errlog] = kmeans(centres, data, options)`
also returns the cluster number (in a one-of-N encoding) for each data
point in `post` and a log of the error values after each cycle in
`errlog`.
The optional parameters have the following interpretations.

`options(1)` is set to 1 to display error values; also logs error
values in the return argument `errlog`.
If `options(1)` is set to 0,
then only warning messages are displayed. If `options(1)` is -1,
then nothing is displayed.

`options(2)` is a measure of the absolute precision required for the value
of `centres` at the solution. If the absolute difference between
the values of `centres` between two successive steps is less than
`options(2)`, then this condition is satisfied.

`options(3)` is a measure of the precision required of the error
function at the solution. If the absolute difference between the
error functions between two successive steps is less than
`options(3)`, then this condition is satisfied.
Both this and the previous condition must be
satisfied for termination.

`options(14)` is the maximum number of iterations; default 100.

## Example

`kmeans` can be used to initialise the centres of a Gaussian
mixture model that is then trained with the EM algorithm.

```
[priors, centres, var] = gmmunpak(p, md);
centres = kmeans(centres, data, options);
p = gmmpak(priors, centres, var);
p = gmmem(p, md, data, options);
```

## See Also

`gmminit`, `gmmem`

---

**Pages:**
Index


---

Copyright (c) Ian T Nabney (1996-9)
